# Predictive factors for severity and poor treatment response in children with Evans syndrome: A retrospective cohort study

**DOI:** 10.3389/fimmu.2026.1701492

**Published:** 2026-02-25

**Authors:** Monia Ben Khaled, Marwa Ben Ayed, Zaid Zaroui, Ilhem Ben Fraj, Samia Rekaya, Najla Mekki, Yosra Chebbi, Aicha Ben Taieb, Amani Merdassi, Ikram Zaiter, Takwa Lamouchi, Ridha Kouki, Houda Kaabi, Hamida Slama, Wafa Achour, Mohamed Bejaoui, Fethi Mellouli, Imen Ben-Mustapha, Monia Ouederni

**Affiliations:** 1 Faculty of Medicine of Tunis, University of Tunis El Manar, Tunis, Tunisia; 2 Pediatric Immuno-Hematology Department, Bone Marrow Transplantation Center of Tunis, Tunis, Tunisia; 3 Laboratory Department, Bone Marrow Transplantation Center of Tunis –Laboratory of Child and Immunocompromised Microbiology (LR18ES39), Faculty of Medicine of Tunis, University of Tunis El Manar, Tunis, Tunisia; 4 Laboratory of Transmission, Control and Immunobiology of Infections, LR16IPT02, Institut Pasteur de Tunis, University of Tunis El Manar, Tunis, Tunisia; 5 Department of Immunohematology, National Center of Blood Transfusion, Tunis, Tunisia

**Keywords:** autoimmune cytopenias, Evans syndrome, immune dysregulation, immunosuppressive therapy, pediatrics, predictors, primary immunodeficiencies, survival

## Abstract

Evans syndrome (ES) is a rare disorder characterized by autoimmune cytopenias affecting multiple blood cell lineages. In children, management remains particularly challenging due to the absence of clear guidelines for acute treatment and escalation to second-line therapies. We conducted a retrospective, longitudinal study (2010-2024) including pediatric patients (<18 years) with ES to identify predictors of severe presentation, need for second-line therapy, and fatal outcome. Predictors were identified using Cox Regression. Fifty patients were included (sequential = 27, concomitant = 23), with a median age at diagnosis of 4.1 years (IQR:1.5–8.5). Secondary ES was observed in 41(82%) cases, among which 38 (76%) had IEI. Severe clinical presentation at diagnosis occurred in 50% of patients and was independently associated with age < 24months and hemoglobin level < 80g/L. Corticosteroid dependence was observed in 34 cases (68%), with second-line therapy required in 31 patients (62%, cumulative risk=88%). This was associated with the presence of hepatomegaly and abnormal IgM levels. At last follow-up, 38(76%) patients were in remission and 19(38%) had relapsed. Fatal outcome (8 patients) was associated with age < 24 months at diagnosis (p=0.04), family history of IEI (p=10^-3^), splenomegaly (p=0.02), and hepatomegaly (p=0.05). Pediatric ES is therefore a severe condition particularly in infants. Outcomes are strongly influenced by underlying immune dysregulation, highlighting the need for early etiological evaluation to guide timely and appropriate therapeutic strategies including the early use of targeted or curative approaches.

## Graphical Abstract

**Figure:**
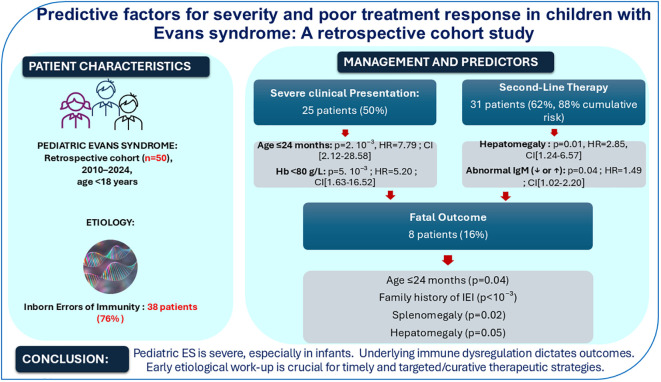

## 1 Introduction

Evans syndrome (ES) is a rare and heterogeneous autoimmune disorder defined by the coexistence or sequential occurrence of immune cytopenias, most commonly autoimmune hemolytic anemia (AIHA) and immune thrombocytopenia (ITP), and less frequently autoimmune neutropenia (1, 2). The pathogenesis of ES reflects immune dysregulation driven by both genetic susceptibility and acquired immune imbalance. This includes impaired regulatory T-cell (Treg) function, leading to defective peripheral tolerance and B-cell hyperactivity with autoantibody production (3). Environmental factors, such as infections or inflammatory immune activation, are thought to act as triggering events in genetically predisposed individuals (2). In pediatric cases, secondary forms are more common with nearly 60% associated with inborn errors of immunity (IEI), predominantly within diseases of immune dysregulation category (2). In contrast, adult ES more often presents as a primary condition, with more heterogeneous etiologies and fewer identifiable monogenic causes (4).

The heterogeneous presentation and unpredictable course of pediatric ES pose substantial diagnostic and therapeutic challenges. Evidence-based treatment guidelines remain limited for this population (1). Corticosteroids are widely used as first-line therapy, but in cases of ES secondary to IEI, management often requires disease-modifying approaches (e.g. abatacept) or even curative hematopoietic stem cell transplantation (5), depending on the underlying diagnosis. Nevertheless, relapse rates are high, and treatment responses are not yet fully elucidated (6). Identifying predictors of severe disease and poor response is therefore critical to recognize patients at higher risk of unfavorable outcomes and to tailor therapeutic strategies in a timely manner.

This retrospective study aimed to identify predictors of severe presentation or poor therapeutic response in children with ES.

## 2 Materials and methods

### 2.1 Design of study

This was a 15-year retrospective longitudinal study (2010–2024) conducted in the Department of Pediatrics, Immuno-Hematology and Stem Cell Transplantation at the National Bone Marrow Transplantation Center, Tunisia.

### 2.2 Study population

This study included pediatric patients (<18years at diagnosis) who fulfilled the diagnostic criteria for ES, defined as concurrent or sequential AIHA and ITP, with or without neutropenia (7). Exclusion criteria were ES occurring after hematopoietic stem cell transplantation and cases with a negative direct antiglobulin test (DAT).

AIHA was defined by clinical and laboratory evidence of hemolysis with a positive DAT. ITP was defined as isolated thrombocytopenia (platelet count<100 G/L, confirmed by peripheral blood smear) with no identifiable underlying cause (8).

The diagnosis and classification of IEI were based on the 2019 European Society for Immunodeficiencies (ESID) diagnostic criteria and the 2022 International Union of Immunological Societies (IUIS) updated classification (9, 10).

### 2.3 Data collection

Data were recorded from patient’s medical records. It included epidemiological characteristics, clinical, biological, therapeutic, and evolutionary features.

Clinical and laboratory evaluations included the following:

- Detailed clinical assessment to identify signs suggestive of IEI, including family and personal history, recurrent infections, allergies, adverse post-vaccination reactions, chronic diarrhea and the presence of hepatosplenomegaly or lymphadenopathy. Chronic lymphoproliferation was defined as lymphadenopathy and/or hepatosplenomegaly persisting for more than six months in the absence of infection or malignancy.
- Systematic infectious screening, including PCR and/or serological testing for *Mycoplasma pneumoniae*, hepatitis B and C viruses, Epstein–Barr virus, cytomegalovirus, HIV-1, parvovirus B19, adenoviruses, and herpesviruses (HSV 1/2/6/8). Additional pathogen-specific testing was performed based on clinical presentation or epidemiologic exposure.
- Autoimmune and serologic testing, including antinuclear antibodies (titer >1:80), anti–double-stranded DNA antibodies, rheumatoid factor (>20IU/mL), thyroid autoantibodies with free thyroxine and TSH measurements, diabetes-related autoantibodies (GAD, islet cell, IA-2, and insulin antibodies), markers of autoimmune hepatitis (ANA, anti–liver-kidney microsome type 1, anti-smooth muscle, and anti–soluble liver antigen antibodies), and celiac disease screening (IgA anti-tissue transglutaminase antibodies, or IgG in IgA-deficient patients).
- Hematologic evaluation, including peripheral blood smear and bone marrow aspiration, with optional immunophenotyping and karyotype analysis.
- Immunologic profiling including plasma protein electrophoresis, quantitative immunoglobulin levels (IgG, IgA, IgM, IgE), lymphocyte subset analysis (CD3, CD4, CD8, CD19, CD56, HLA-DR and double-negative T cells [CD3+CD4–CD8–TCRαβ]), lymphocyte proliferation assays, and nitroblue tetrazolium testing in patients with suspected phagocytic defects.
- Genetic testing including targeted gene panels or whole-exome sequencing via next-generation sequencing for selected patients with clinical suspicion or refractory disease, when financially feasible.

Evans syndrome was considered to be diagnosed at the time of the second cytopenia. It was classified as secondary when an identifiable underlying condition was identified, including IEI, systemic autoimmune diseases, hematological malignancies or infections. It was classified as primary when no cause was found after complete evaluation including immunological and genetic investigations (11).

Clinical severity at diagnosis related to cytopenia was defined by the occurrence of: persistent mucosal bleeding requiring medical intervention to stop hemorrhage, deep visceral bleeding, hemorrhagic shock, poorly tolerated anemia leading to impaired general condition, marked asthenia, tachycardia, dyspnea, or heart failure, febrile neutropenia with sepsis or septic shock.

Corticosteroid sensitivity was classified as follows:

-Complete Remission: Hemoglobin normal for age, reticulocyte count <120G/L and platelets >100G/L without bleeding. No clinical or laboratory signs of hemolysis (no jaundice, normal urine color, haptoglobin >0.3g/L, indirect bilirubin <17µmol/L, Lactate dehydrogenase <250IU/L).
-Partial Remission: Hemoglobin 70g/L to normal for age, reticulocyte count >120G/L without transfusion requirements, and platelets 20–100 G/L without bleeding. Hemolysis markers within normal limits as above.
-Corticodependence: Relapse of cytopenia during steroid tapering or after discontinuation.
-Corticoresistance: No remission after four weeks of oral steroids and three IV methylprednisolone pulses (1 g/1.73 m², 48h apart).
-Relapse: Recurrence of hemolysis or bleeding after achieving complete or partial remission, whether on or off treatment.
-Failure to respond to initial treatment and the need for second-line therapy were defined as corticosteroid resistance or dependence on high-dose corticosteroids (dose > 0.2 mg/kg/day).

### 2.4 Statistical method

Statistical analyses were performed using SPSS version 24. Cumulative incidence of severe presentation at diagnosis was analyzed using the log-rank test in a univariate analysis. The time origin was defined as the date of first symptoms, and the event was defined as the date of onset of severity signs related to cytopenias. The cumulative incidence of the need of second-line therapy, and death was estimated by Kaplan–Meier method with the time origin set at the date of diagnosis. Group comparisons were performed using the log-rank test in univariate analysis. Hemoglobin and platelet counts were dichotomized into categorical variables using ROC curve analysis. Variables with p <0.10 in univariate analysis were entered into a multivariate cox regression model to identify independent predictors. In accordance with the rule of 10 events per variable, the multivariable models were restricted to the most clinically relevant variables that were also significantly associated with the event. Statistical significance was defined as p <0.05.

### 2.5 Compliance with ethical standards

The study was approved by the local ethics committee [Ref.number: CNGMOEC-0424], with access to patient records granted without consent due to its retrospective design and anonymized data (no intervention or modification of patient care). Informed consent was obtained prior to genetic testing.

## 3 Results

### 3.1 Demographics and baseline characteristics

A total of 50 patients were diagnosed with ES during the study period, with a male-to-female ratio of 1.17. The initial cytopenia occurred at a median age of 3.3 years (IQR: 1.1–6.8), while the median age at ES diagnosis was 4.1 years (IQR: 1.5–8.5). Consanguinity was present in 23 patients (46%), and a family history of immuno-hematological disorders was reported in 15 cases (30%).

Clinical examination identified features suggestive of IEI in 28 patients (56%), irrespective of cytopenia-related manifestations. The most frequent clinical findings included chronic lymphoproliferation (21 patients; 42%), recurrent infections (8 patients; 16%), diarrhea (6 patients; 12%), eczema (3 patients; 6%), and pulmonary micronodules (3 patients; 6%). Myopathy, nodular spleen and renal insufficiency were each observed in one patient (2%).

Cytopenias occurred sequentially in 27 cases (54%) and simultaneously in 23 cases (46%) with a median interval of 12.9 months (IQR: 5.6–51) between the first and second cytopenia. The initial cytopenia was ITP in 15 patients (30%) and AIHA in 12 patients (24%).

Cytopenia-related severity signs were observed in 25 patients (50%), with a cumulative risk of 85% from the onset of first clinical manifestations to diagnosis. Twelve patients (24%) exhibited features of poor anemia tolerance, including tachycardia (n=8), tachypnea (n=6), and shock with altered consciousness (n=2). Severe bleeding occurred in 14 patients (28%), involving mucosal or gastrointestinal sites (n=10), intracranial (n=5), retinal (n=2), or menorrhagia (n=2). Patients who experienced bleeding had a mean platelet count of 11.2 G/L versus 76.8 G/L in those without bleeding (p=0.02), with a cutoff of 20 G/L according to ROC curve analysis.

At the time of ES diagnosis, 24 patients (48%) had bicytopenia (AIHA and thrombocytopenia) and 9 (18%) had pancytopenia. Single cytopenia was noted in 26 patients including 11 cases of isolated AIHA and 15 cases of isolated thrombocytopenia. Among the 35 patients with AIHA at diagnosis, 11 had a reticulocyte count below 120 G/L. Baseline hematologic and immunologic findings are summarized in **Table 1**.

**Table 1:** Summary of biological findings in 50 pediatric patients with Evans syndrome.

Parameter	Median (Q1-Q3)
Hemogram	
Hemoglobin (g/L)	79 (59-105)
Mean corpuscular volume (Fl)	78.0 (72.0-91.0)
Platelet count (G/L)	17.0 (4.5-46.0)
Neutrophil (G/L)	3.2 (0.5-5.0)
Lymphocytes (G/L)	2.1 (1.6-4.4)
Reticulocyte count (G/L)	151.0 (66.2-295.3)
Lactate dehydrogenase (U/l)	295.0 (267.7-556.2)
Total bilirubin (µmol/l)	8.0 (5.0-12.0)
Direct bilirubin (µmol/l)	3.0 (2.25 -3.0)
Haptoglobin (g/l)	0.21 (0.02 -1.11)
Gamma globulins (g/l)	18.7 (10.45-23.65)
Immunoglobulins (g/l)	
IgG(g/l)	11.55 (6.94-16.17)
IgM(g/l)	0.86 (0.54-1.50)
IgA(g/l)	0.97 (0.44-1.69)
Lymphocytes phenotyping (%)	
CD3(%)	75.0 (67.0-82.0)
CD4(%)	34.0 (25.0-50.0)
CD8(%)	27.0 (21.0-39.0)
CD56(%)	6.3 (2.4-7.8)
CD19(%)	10.5 (4.5-17.4)
CD3^+^CD4^-^CD8^-^ (Double-negative) T Cells(%)	1.8 (1.13-5.4)

Peripheral blood smears revealed anisocytosis in 26 cases (52%), activated lymphocytes in 6(12%), blasts in 1(2%), rare platelets in 23 (46%) and microplatelets in 2(4%). Direct antiglobulin testing identified warm-reactive antibodies in 48 patients (96%), isolated cold agglutinins in one patient (2%) and biphasic antibodies in one patient (2%).

Other autoimmune manifestations were observed in 8 patients (16%), including celiac disease (n=4), type 1 diabetes with anti-GAD antibodies (n=2), autoimmune thyroiditis (n=1), systemic lupus erythematosus (n=1), and positive ANA/anti-DNA antibodies (n=2).

Whole-exome sequencing was performed in 20 patients, while targeted gene panels were performed in two additional patients. Mutations suggestive of an IEI were identified in 18 cases, whereas results were negative in the remaining four.

### 3.2 Etiologies and management of Evans Syndrome

**Figure 1:**
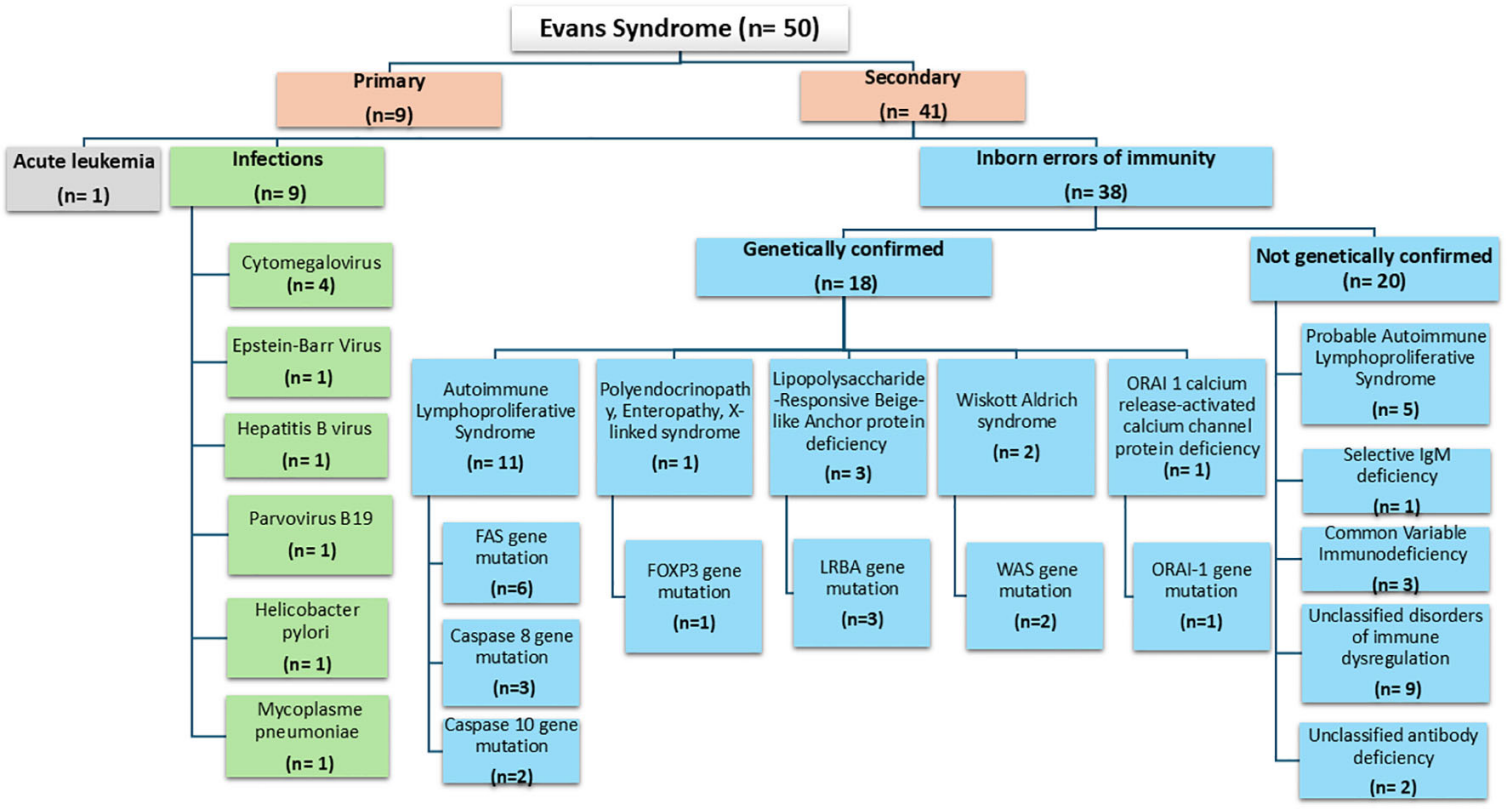
Etiologies of 50 pediatric patients with Evans syndrome. An underlying inborn error of immunity was identified in 38 patients (76%). Infectious triggers were identified in 9 patients (18%); notably, six patients presented with dual etiologies, combining an underlying IEI and a concomitant infectious trigger.

Evans syndrome was classified as secondary in 41 cases (82%), while the etiology remained undetermined in nine cases (18%). An underlying IEI was identified in 38 patients (76%), predominantly within the diseases of immune dysregulation group (n=32; 64%), with confirmed or probable ALPS in 16 cases (32%). Infectious causes were identified in 9 patients (18%), including six viral infections. Among these, six patients had both an underlying IEI and a concomitant infectious trigger. The distribution of etiologies is illustrated in **Figure 1**.

All patients received corticosteroids as first-line therapy, and 30 patients (60%) were also treated with intravenous immunoglobulin (1 g/kg/dose). Thirty-one patients (62%) required second-line treatment, with a cumulative incidence of 88%. Mycophenolate mofetil was the most frequently used second-line agent (n=17), followed by azathioprine (n=7) and rapamycin (n=7). Additional therapies included splenectomy (n=4), cyclosporine (n=1), vincristine (n=2), and rituximab (n=1). Four patients underwent hematopoietic stem cell transplantation.

**Figure 2:**
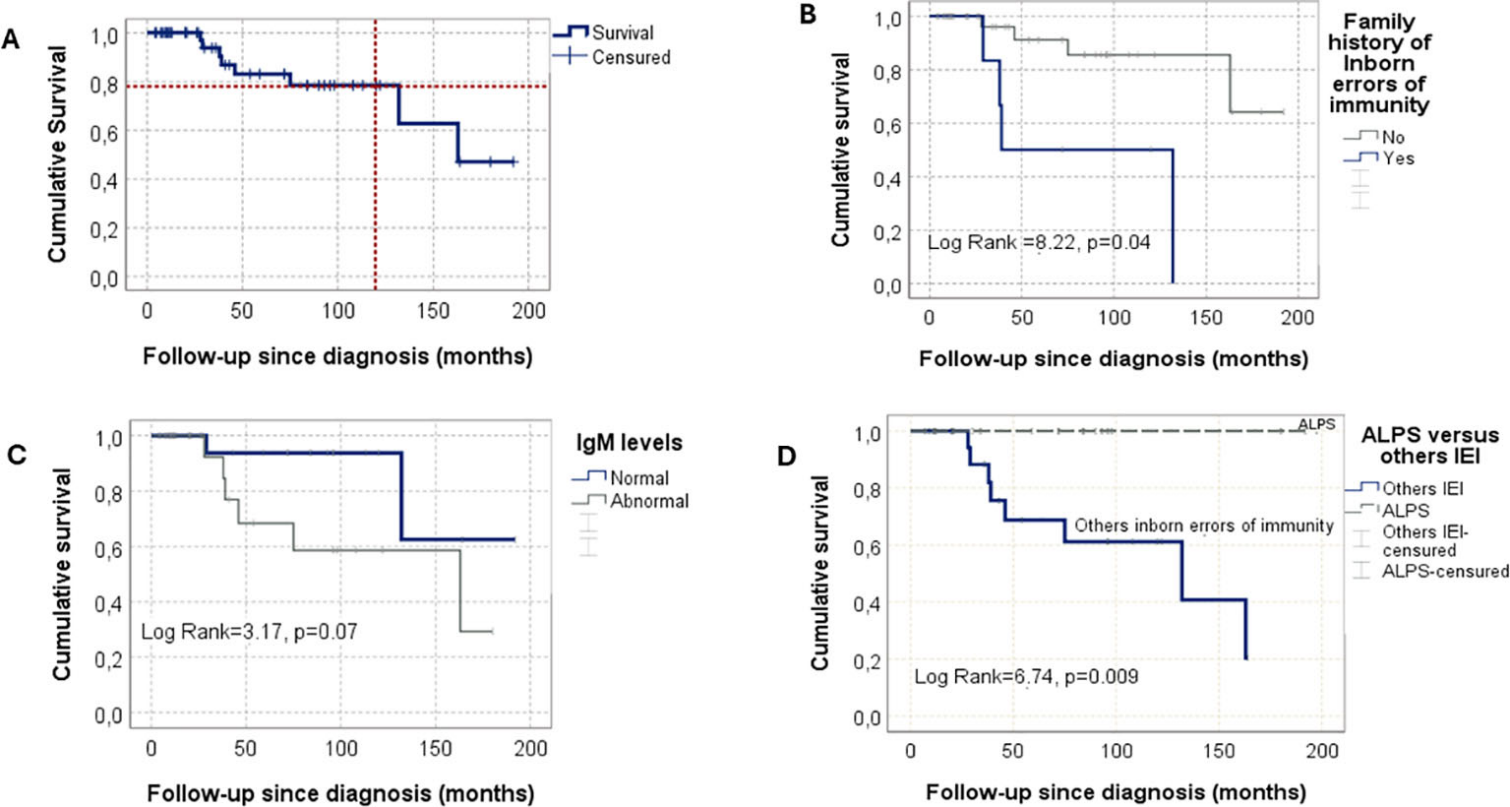
Overall survival in children with Evans syndrome (n = 50). **(A)** Overall survival: 83% at 5 years, 78% at 10 years, and 47% at 15 years of follow-up. **(B)** Overall survival according to the presence or absence of clinical features suggestive of an inborn error of immunity. **(C)** Overall survival according to abnormal (either decreased or elevated) or normal IgM levels. **(D)** Overall survival according to ES etiology: ALPS vers other IEI. IEI, inborn errors of immunity; ALPS, autoimmune lymphoproliferative syndrome.

After a median follow-up of 114 months (IQR: 60–193), remission was achieved in 38 patients (76%), of whom 23 remained on immunosuppressive therapy. Fourteen patients (28%) achieved complete remission, and 24 (48%) partial remission. Nineteen patients (38%) experienced at least one relapse, with a median of four episodes (IQR:3–8). During follow-up, 42 patients (84%) survived, while eight (16%) died. Cumulative overall survival rates were 83% at 5 years, 78% at 10 years, and 47% at 15 years (**Figure 2**). Causes of death included severe cytopenias (n=3), infections (n=2), end-stage renal failure (n=1), and complications related to hematopoietic stem cell transplantation (n=2).

### 3.3 Risk factors of severe presentation or of poor initial and final therapeutic response

**Figure 3:**
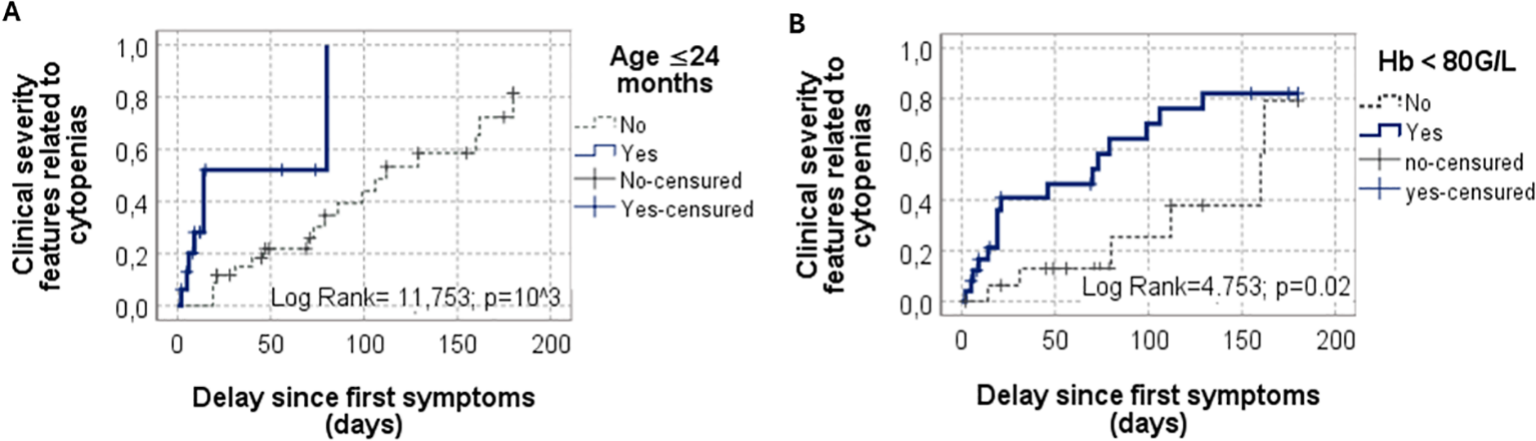
Clinical Severity Features Related to Cytopenias in children with Evans syndrome (n=50). Kaplan–Meier survival analysis of time from first symptoms to the occurrence of clinical severity features, stratified by: **(A)** Age ≤24 months (Log Rank=11.753; p<0.001). **(B)** Hemoglobin <80 g/L (Log Rank=4.753; p=0.02).

Results of univariate and multivariable analyses are summarized in **Tables 2**, **3**. At diagnosis, clinical presentation was significantly more severe in infants (p=10^-3^) and in patients with hemoglobin levels below 80 g/L (p=0.02); both factors remained significant in multivariable Cox regression analysis (**Figure 3**).

**Table 2:** Predictors of clinical severe presentation at diagnosis (25 out of 50 children with Evans syndrome).

Parameters	Univariate analysis					Multivariable analysis			
	At risk categories	Cumulative risk %	Control category	Cumulative risk %	p-value	Beta coefficient	p-value	Hazard Ratio	95% confidential interval
Gender (female=23)	Yes	90	No	73	0.84	–	–	–	–
Age ≤24 months (n=16)	**Yes**	**100**	No	82	**10^-3^**	**2.05**	**2.10^-3^**	**7.79**	**2.12-28.58**
Consanguinity (n=23)	No	80	yes	70	0.50	–	–	–	–
Family history of IEI (n= 8)	Yes	100	No	76	0.06	1.00	0.08	2.72	0.87-8.51
Clinical signs suggestive of IEI (n=28)	Yes	84	No	57	0.83	–	–	–	–
Presence of a triggering factor (n=10)	No	83	Yes	38	0.61	–	–	–	–
Splenomegaly (n=30)	Yes	81	No	63	0.96	–	–	–	–
Hepatomegaly (n=19)	Yes	89	No	78	0.29	–	–	–	–
Peripheral lymphadenopathy (n=21)	No	87	Yes	66	0.34	–	–	–	–
Initial thrombocytopenia (n=15)	No	88	yes	76	0.06	–	–	–	–
Initial autoimmune hemolytic anemia (n=12)	Yes	86	No	88	0.69	–	–	–	–
Concomitant cytopenias(n=23)	No	89	Yes	79	0.29	–	–	–	–
Platelet count <20 G/L (n=30)	Yes	84	No	52	0.51	–	–	–	–
Hemoglobin level ≤ 80 g/L	**Yes**	**83**	No	80	**0.02**	**1.65**	**5.10^-3^**	**5.20**	**1.63-16.52**
Neutropenia (n=9)	Yes	82	No	66	0.31	–	–	–	–
Hypergammaglobulinemia (n=15)	Yes	100	No	85	0.81	–	–	–	–
Abnormal IgG levels (n=26)	Yes	82	No	56	0.67	–	–	–	–
Abnormal IgM levels (n=15)	Yes	89	No	65	0.49	–	–	–	–
Abnormal IgA levels (n=13)	Yes	86	No	54	0.18	–	–	–	–
Secondary Evans syndrome(n=40)	Yes	85	No	47	0.39	–	–	–	–
Underlying inborn errors of immunity (n=38)	Yes	84	No	60	0.66	–	–	–	–
Autoimmue lymphoproliferative syndrome (n=16)	No	91	Yes	65	0.87	–	–	–	–

Bold characters indicate statistically significant associations.

**Table 3:** Predictors of the need of second-line therapy (31 out of 50 children with Evans syndrome).

Parameters	Univariate analysis					Multivariable analysis			
	At risk categories	Cumulative risk %	Control category	Cumulative risk %	p-value	Beta coefficient	p-value	Hazard Ratio	95% confidential interval
Gender (female=23)	Yes	79	No	74	0.76	–	–	–	–
Age ≤24 months (n=16)	Yes	100	No	85	0.41	–	–	–	–
Consanguinity (n=23)	No	84	yes	70	0.73	–	–	–	–
Family history of IEI (n= 8)	Yes	100	No	85	**0.05**	0.62	0.20	1.86	0.71-4.82
Clinical signs suggestive of IEI (n=35)	Yes	90	No	84	0.67	–	–	–	–
Presence of a triggering factor (n=10)	No	100	Yes	66	0.57	–	–	–	–
Splenomegaly (n=30)	Yes	87	No	77	**0.01**	–	–	–	–
Hepatomegaly (n=19)	Yes	100	No	82	**0.02**	**1.05**	**0.01**	**2.85**	**1.24-6.57**
Peripheral lymphadenopathy (n=21)	No	87	Yes	78	0.92	–	–	–	–
Clinical severity related to cytopenias (n=25)	Yes	91	No	87	0.57				
Initial thrombocytopenia (n=15)	No	91	yes	81	0.70	–	–	–	–
Initial autoimmune hemolytic anemia (n=12)	Yes	84	No	74	0.85	–	–	–	–
Concomitant cytopenias(n=23)	No	82	Yes	64	0.84	–	–	–	–
Sequential onset of cytopenias or tricytopenia at diagnosis (n=35)	Yes	85	No	62	0.81	–	–	–	–
Platelet count <20 G/L (n=30)	No	52	Yes	28	0.10	–	–	–	–
Hemoglobin level ≤ 80 g/L	Yes	90	No	62	0.23	–	–	–	–
Neutropenia (n=9)	No	88	Yes	74	0.92	–	–	–	–
Hypergammaglobulinemia (n=15)	No	85	Yes	76	0.95	–	–	–	–
Abnormal IgG levels (n=26)	Yes	90	No	87	0.23	–	–	–	–
Abnormal IgM levels (n=15)	Yes	100	No	85	0.09	**0.4**	**0.04**	**1.49**	**1.02-2.20**
Abnormal IgA levels (n=13)	No	91	Yes	79	0.47	–	–	–	–
Secondary Evans syndrome(n=40)	Yes	90	No	70	0.13	–	–	–	–
Underlying inborn errors of immunity (n=38)	Yes	93	No	58	0.25	–	–	–	–
Autoimmue lymphoproliferative syndrome (n=16)	No	100	Yes	94	0.69	–	–	–	–

Bold characters indicate statistically significant associations.

**Figure 4:**
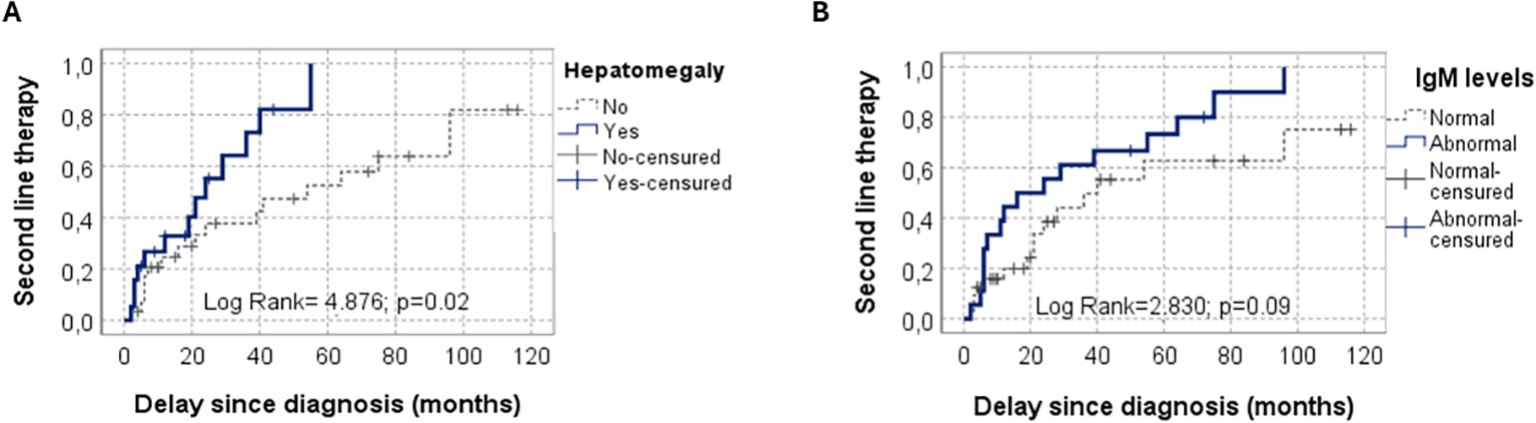
Predictors of Need for Second-Line Therapy in children with Evans syndrome (n=50). Kaplan–Meier curves showing time from diagnosis of Evans syndrome to initiation of second-line therapy, stratified by: **(A)** Presence of hepatomegaly (Log Rank=4.876; p=0.02). **(B)** Abnormal (either decreased or elevated) or normal IgM levels (Log Rank=2.830; p=0.09).

The need for second-line therapy was associated with a family history of IEI (p=0.05), splenomegaly, and hepatomegaly on physical examination (p=0.01 and 0.02, respectively). In multivariable analysis, hepatomegaly and abnormal IgM levels (either decreased or elevated) independently predicted first-line treatment failure (**Figure 4**).

At last follow-up, fatal outcomes were associated with age <24 months at diagnosis (p=0.04), family history of IEI (p=10^-3^), splenomegaly (p=0.02), and hepatomegaly (p=0.05). Conversely, ALPS diagnosis (p=0.009) and treatment with MMF or rapamycin (p=0.01) were associated with improved survival (**Table 4**).

**Table 4:** Univariate analysis of factors associated with fatal outcome in 8 of 50 children with Evans syndrome.

Parameters	At risk categories	Cumulative risk %	Control category	Cumulative risk %	p-value
Gender (female=23)	Yes	54	No	32	0.38
Age ≤24 months (n=16)	Yes	60	No	48	**0.04**
Consanguinity (n=23)	No	100	yes	37	0.11
Family history of IEI (n= 15)	Yes	100	No	56	**10-3**
Clinical signs suggestive of IEI (n=28)	Yes	56	No	0	0.23
Presence of a triggering factor (n=10)	No	62	Yes	17	0.64
Splenomegaly (n=30)	No	45	Yes	43	**0.02**
Hepatomegaly (n=19)	Yes	40	No	32	**0.05**
Peripheral lymphadenopathy (n=21)	No	100	Yes	35	0.14
Clinical severity related to cytopenias (n=25)	Yes	76	No	17	0.22
Initial thrombocytopenia (n=15)	No	67	yes	43	0.11
Initial autoimmune hemolytic anemia (n=12)	Yes	47	No	62	0.43
Concomitant cytopenias(n=23)	No	57	Yes	10	0.33
Abnormal IgG levels (n=26)	Yes	54	No	20	0.96
Abnormal IgM levels (n=15)	Yes	71	No	38	0.07
Abnormal IgA levels (n=13)	No	55	Yes	33	0.33
Secondary Evans syndrome(n=40)	Yes	64	No	0	0.48
Underlying inborn errors of immunity (n=38)	Yes	54	No	0	0.48
Initial steroid resistance or dependance (n=34)	Yes	55	No	15	0.64
Mycophenolate mofetil or Rapamycin (n=21)	No	54	Yes	77	**0.01**
Underlying inborn errors of immunity (n=38)	Yes	93	No	58	0.25
Autoimmue lymphoproliferative syndrome(n=16)	No	80	Yes	0	**9.10^-3^**

Bold characters indicate statistically significant associations.

## 3 Discussion

This retrospective descriptive and analytical study included 50 pediatric patients diagnosed with ES who were followed at a Tunisian reference center over a 15-year period. The study identified predictors of acute severe presentations, the need for second-line therapy and fatal outcomes. A high prevalence of IEI was observed (76%), predominantly within the diseases of immune dysregulation group (64%). A substantial proportion of patients exhibited corticosteroid resistance or dependence (68%).

Although ES manifests primarily as peripheral cytopenias, clinical signs of severity were present at diagnosis in half of the patients. Severe manifestations were significantly associated with infant age (p=2.10^-3^; HR = 7.79, 95% CI:2.12–28.58) and with a hemoglobin level **≤**80 g/L (p=5.10^-3^; HR = 5.20, 95% CI:1.63–16.52). In a large French cohort of 156 children, severe presentations were reported in 41/102 (40%) ITP patients and in 65/111(59%) AIHA patients (12). The rapid and profound drop in hemoglobin may lead to significant tissue hypoxia, particularly before effective bone marrow compensation has occurred. The frequent coexistence of severe thrombocytopenia in ES may further increase the risk of hemorrhage contributing to clinical severity.

Currently, no Cochrane review specifically defines transfusion thresholds for pediatric AIHA or ES. Therefore, transfusion decisions are guided by clinical judgment rather than hemoglobin levels alone. In critically ill children with acute brain injury, red blood cell transfusion is often considered when hemoglobin levels range between 70 and 100 G/L (13, 14); a similar approach could be considered in ES.

In this cohort, severe mucosal or visceral bleeding occurred in 28% of patients, including five cases of intracranial hemorrhage. Multiple studies have reported that patients with ES often present with major bleeding episodes, particularly mucosal or visceral (12, 15–17). Hemorrhagic risk was associated with platelet counts **<**20 G/L based on ROC curve analysis, although similar counts are not predictive of bleeding in isolated ITP (6, 8, 18, 19). Several hypotheses may explain the distinct bleeding phenotype observed in ES. First, the intense autoantibody production could impair megakaryopoiesis by targeting bone marrow megakaryocytes, leading to both peripheral platelet destruction and suppressed platelet production. This hypothesis is supported by the poor bone marrow regeneration observed in 11 out of 35 patients with AIHA at diagnosis and could plausibly extend to the platelet lineage. Second, autoimmune-mediated vascular injury may increase vascular fragility and permeability, predisposing to bleeding (6). Third, the hemolytic process itself in AIHA can induce endothelial dysfunction through nitric oxide depletion, which could increase the risk of mucosal or visceral hemorrhage (20). Additional studies are necessary to confirm these hypotheses.

Evans syndrome requires a distinct therapeutic approach from isolated ITP due to its severe and unpredictable course. Acute management combines corticosteroids with intravenous immunoglobulins to achieve a rapid platelet increase and mitigate hemorrhagic risk (16). Long-term management focuses on controlling the underlying autoimmune process to prevent relapse rather than solely managing acute episodes (12).While corticosteroids are a cornerstone of initial therapy for their broad immunosuppressive effect (7, 16), their long-term efficacy is limited and relapses are frequent (12, 21). In our cohort, 31 (62%) required second-line therapy with a cumulative incidence reaching 88%. Similarly, second-line therapy was required for 69% of children (108/156) in a large prospective study (12), and for 63% in a Brazilian cohort (22).

Identifying predictive factors for corticosteroid response is critical to anticipate poor outcome and guide further etiological investigation and alternative treatment. In this cohort, the need for second-line therapy was independently associated with, hepatomegaly and abnormal IgM levels (either decreased or elevated), reflecting the presence of an underlying IEI. Fatal outcome was associated with hepatosplenomegaly or a family history of IEI in univariate analysis. Recent pediatric ES studies have demonstrated that underlying IEI was associated with reduced rates of sustained remission and a greater risk of severe complications and mortality (12, 23). In the Turkish Registry of Immunodeficiencies, which included 34 patients with autoimmune cytopenias, those with diseases of immune dysregulation were significantly more likely to develop resistance to first-line therapies compared to other IEI groups (p=0.021) (24).

An abnormal IgM level is a strong indicator of an underlying IEI, potentially reflecting defective T-B cell cooperation. Low IgM is common in conditions like common variable immunodeficiency or ES-associated syndromes such as LRBA or CTLA4 mutations (23). Hyper-IgM should prompt consideration of Hyper-IgM syndrome, typically characterized by elevated IgM levels with concomitant reduction of other immunoglobulin classes. It is important to exclude false IgG normalization due to prior immunoglobulin replacement therapy, which may mask both IgG deficiency and an underlying Hyper-IgM syndrome. Classical Hyper-IgM syndromes result from mutations in CD40LG, CD40, AICDA, UNG, IKBKG, or PIK3CD/PIK3R1, and less frequently from variants in TNFRSF13B, TNFRSF13C, ICOS, CD19, LRBA, NFKB, VAV1, PLCG2 or other yet unidentified genes (25).

Diseases of immune dysregulation were the most frequently observed IEI group in this cohort, (64%). They present with a broad and heterogeneous clinical spectrum, including cytopenias, enteropathy, lymphoproliferation, or early-onset autoimmunity, which pose substantial diagnostic challenges. Standard immunological workup may be normal or only mildly altered. Whole-exome sequencing is therefore strongly recommended to identify causative mutations, and refine diagnosis, and guide a more personalized therapeutic approach (6, 26).

Early consideration of targeted second-line therapy is indicated in specific situations: the mTOR inhibitor rapamycin in ALPS; abatacept for autoimmune cytopenias due to CTLA-4 haploinsufficiency or LRBA deficiency; JAK inhibitors for STAT3 gain-of-function mutations and selective PI3K delta inhibitors, for activated PI3K delta syndrome (2, 16, 27).

ALPS was the most common identified cause of ES, consistent with the literature (3, 28–30). It was associated with improved overall survival in univariate analysis, as was treatment with rapamycin or mycophenolate mofetil. The efficacy of these therapies in managing ALPS-associated autoimmunity was established, particularly when splenectomy and rituximab are avoided (31, 32). However, a persistent risk of lymphoma remains (28).

This study highlights the severity of pediatric ES, both in its acute presentation and in long-term management. Cytopenias are more severe than in isolated autoimmune cytopenias. AIHA and autoimmune thrombocytopenia may exacerbate each other in a vicious cycle, justifying an aggressive treatment approach during the acute phase to improve cytopenias, particularly in infants. Factors suggestive of an underlying IEI were predictive of both poor initial response and associated with unfavorable long-term outcomes. Consequently, beyond standard immunological workup, early and systematic investigation using next-generation sequencing is essential to rapidly adapt therapy toward targeted interventions or allogeneic stem cell transplantation.

The interpretation of these findings should consider several limitations. The sample size and the risk of overfitting limited the feasibility and statistical power of multivariate analyses, precluding a reliable multivariable evaluation of predictors of fatal outcomes. In addition, the long recruitment period may have introduced heterogeneity due to evolving etiological classification and therapeutic practices, with limited availability of advanced genetic testing early in the cohort. Finally, the retrospective design and recruitment from a reference immunohematology center may have introduced selection toward more complex cases. Nonetheless, the relatively large cohort size and long-term follow-up provide valuable insights into pediatric ES and allow identification of clinically meaningful predictors of poor outcomes.

In conclusion, ES in pediatric patients is a complex and often chronic condition, frequently associated with underlying IEI. In the acute phase, half of the patients presented with cytopenia-related severity signs, particularly in infants and in those with hemoglobin levels below 80 g/L. Poor response to first-line therapy, as well as unfavorable long-term outcomes, were independently associated with clinical or biological features suggestive of an underlying IEI.

## Data Availability

The original contributions presented in the study are included in the article/supplementary material. Further inquiries can be directed to the corresponding author/s.
